# A vibrotactile behavioral battery for investigating somatosensory processing in children and adults

**DOI:** 10.1016/j.jneumeth.2013.04.012

**Published:** 2013-05-06

**Authors:** Nicolaas A.J. Puts, Richard A.E. Edden, Ericka L. Wodka, Stewart H. Mostofsky, Mark Tommerdahl

**Affiliations:** a Russell H. Morgan Department of Radiology and Radiological Science, The Johns Hopkins University School of Medicine, 600 North Wolfe Street, Baltimore, MD 21287, USA; b F.M. Kirby Center for Functional Brain Imaging, Kennedy Krieger Institute, 707 North Broadway Street, Room G-25, Baltimore, MD 21205, USA; c Center for Autism and Related Disorders, Kennedy Krieger Institute, 3901 Greenspring Avenue, Baltimore, MD 21211, USA; d Department of Neurology, Johns Hopkins University, School of Medicine, 600 North Wolfe Street, Baltimore, MD 21287, USA; e Department of Psychiatry and Behavioral Sciences, Johns Hopkins School of Medicine, 600 North Wolfe Street, Baltimore, MD 21287, USA; f Laboratory for Neurocognitive and Imaging Research, Kennedy Krieger Institute, 716 N Broadway, Baltimore, MD 21205, USA; g Department of Biomedical Engineering, University of North Carolina at Chapel Hill, Chapel Hill, NC 27599, USA

**Keywords:** Vibrotactile, Behavioral, GABA, Pediatric, Somatosensory, Stimulator

## Abstract

The cortical dynamics of somatosensory processing can be investigated using vibrotactile psychophysics. It has been suggested that different vibrotactile paradigms target different cortical mechanisms, and a number of recent studies have established links between somatosensory cortical function and measurable aspects of behavior. The relationship between cortical mechanisms and sensory function is particularly relevant with respect to developmental disorders in which altered inhibitory processing has been postulated, such as in ASD and ADHD. In this study, a vibrotactile battery consisting of nine tasks (incorporating reaction time, detection threshold, and amplitude- and frequency discrimination) was applied to a cohort of healthy adults and a cohort of typically developing children to assess the feasibility of such a vibrotactile battery in both cohorts, and the performance between children and adults was compared. These results showed that children and adults were both able to perform these tasks with a similar performance, although the children were slightly less sensitive in frequency discrimination. Performance within different task-groups clustered together in adults, providing further evidence that these tasks tap into different cortical mechanisms, which is also discussed. This clustering was not observed in children, which may be potentially indicative of development and a greater variability. In conclusion, in this study, we showed that both children and adults were able to perform an extensive vibrotactile battery, and we showed the feasibility of applying this battery to other (e.g., neurodevelopmental) cohorts to probe different cortical mechanisms.

## 1. Introduction

The cortical dynamics of somatosensory processing can be investigated using vibrotactile psychophysics. It has been suggested that different vibrotactile paradigms target different cortical mechanisms, and a number of recent studies have established links between somatosensory cortical function and measurable aspects of behavior (Hernandez et al., 2000; Puts et al., 2011; Romo et al., 2003). However, links between GABAergic inhibitory neurotransmission and behavioral measures are less well understood. GABAergic inhibition is important in shaping the neuronal response to sensory stimulation (Alloway and Burton, 1986; Dykes et al., 1984; Juliano et al., 1989), and most vibrotactile tasks rely in part on cortical GABAergic inhibitory mechanisms (Tommerdahl et al., 2010). Recent developments have made it possible to measure neurotransmitter concentration noninvasively in humans and correlate these concentrations with measures of tactile sensitivity (e.g., Puts et al., 2011).

The relationship between GABA and sensory function is particularly relevant with respect to developmental disorders in which altered GABAergic processing has been postulated. For example, in Autism Spectrum Disorder (ASD), abnormal cortical structure (Casanova, 2004) and sensory processing (Blakemore et al., 2006; Tommerdahl et al., 2008b) have been linked to GABAergic processing, and GABA-system genetic variants have been proposed as models for ASD (e.g., (DeLorey, 2005)). In Tourette syndrome, both an altered density of GABAergic neurons (Kalanithi et al., 2005) and sensory impairments have been described (Belluscio et al., 2011; Miguel et al., 2000), and GABA gene markers correlate with tic severity (Tian et al., 2011). Finally, GABA reductions have been shown in attention-deficit hyperactivity disorder (ADHD) (Edden et al., 2012), and impaired inhibition during cortical stimulation suggests reduced abnormal GABA interneuron activity (Gilbert et al., 2011). Thus, understanding the differences in sensory processing between groups may allow for a better understanding of cortical (dys)function in health and disease.

In this study, we present a battery of vibrotactile tasks that targeted different aspects of cortical function. We demonstrate their feasibility in healthy adults (HA) and typically developing children (TDC), a prerequisite for future clinical studies, and present normative results. We present these data in the context of previous work in the field (Puts et al., 2011; Tannan et al., 2007a, 2007b; Tommerdahl et al., 2008b; Zhang et al., 2011) (Lee et al., 2012; Nelson et al., 2012; Nguyen et al., 2013a, 2013b; Rai et al., 2012) to compare the performance of children and adults and to investigate patterns of performance. A priori, we would expect absolute levels of performance to differ between the HA and TDC but the relationships between related tasks to be preserved.

### 1.1. Overview of task groups

#### 1.1.1. Reaction time

A simple reaction time experiment (‘press when you feel the stimulus’) is a straightforward task for naïve participantsthat allows them to become familiarized with the vibrotactile stimulation. Reaction time has been closely linked to white matter structure (Kerchner et al., 2012; Tamnes et al., 2012) and GABA concentration (Stagg et al., 2011) in healthy subjects. In addition, reaction time has been shown to be altered in developmental disorders (Debes et al., 2011; Schuerholz et al., 1996; Xiao et al., 2012). Reaction time probes both attentional and sensorimotor components.

#### 1.1.2. Detection threshold

The static detection threshold task is a well-known diagnostic tool. An abnormal detection threshold has been used as an indicator of brain dysfunction (Belluscio et al., 2011; Nudo et al., 2000; Staines et al., 2002) and is dependent on both white matter structure (Mountcastle et al., 1972) and GABAergic mechanisms (DeLorey et al., 2011; Tavassoli et al., 2012). In a static vibrotactile detection threshold experiment, the weakest detectable stimulus is typically determined in either a yes/no or a two-alternative forced-choice (2AFC) manner. In contrast, a dynamic vibrotactile detection threshold experiment consists of a stimulus that is increased until perceived (see Zhang et al., 2011). It is thought that pre-detection sub-threshold stimulation mainly activates local feed-forward inhibitory mechanisms (Blankenburg et al., 2003; Favorov and Kursun, 2011; Middleton et al., 2012; Swadlow, 2003), which thereby raises the detection threshold. Comparing dynamic and static threshold measures probes this feed-forward inhibition.

#### 1.1.3. Amplitude discrimination

Discriminating between two stimuli that are simultaneously applied to adjacent digits engages lateral inhibition to separate the response functions of the cortical areas representing each stimulus. A repetitive or ‘adapting’ stimulus has been shown to sharpen this response function (Whitsel et al., 1989, 2003), either by improving signal-to-noise or spatial resolution. Behaviorally, Hollins and Goble (1993) have shown that single-digit amplitude discrimination is improved by a 5 s adapting stimulus prior to each trial. In a similar fashion, Tannan et al. (2007b) have shown that in a healthy population, dual-site amplitude discrimination is improved when each trial is preceded by dual-site adaptation but is diminished when each trial is preceded by adaptation on only one of the digits. Interestingly, this effect of adaptation is absent in ASD (Tommerdahl et al., 2007).

#### 1.1.4. Frequency discrimination

Discriminating the frequency of two sequentially applied stimuli relies upon temporal processing. McLaughlin and Juliano (2005) showed that frequencies were, at least in part, encoded by the periodic synchronous firing of neuronal ensembles in the primary somatosensory cortex (S1) and that applying a GABA antagonist destroys this periodicity. We have previously shown that individual differences in frequency discrimination performance were correlated with GABA concentration in the sensorimotor cortex, as measured by edited MRS (Puts et al., 2011). In contrast, when frequencies are applied simultaneously to adjacent digits, temporal synchronization between the cortical areas, mediated by GABAergic lateral inhibition, would be expected to disrupt the temporal and periodic encoding of each stimulus, thereby impairing discrimination (e.g., Tommerdahl et al., 2008b).

## 2. Materials and methods

### 2.1. Participants

Two cohorts were tested on a tactile battery consisting of nine tasks. Thirteen healthy adults (aged 30.5 ± 4.9 years old; 3 female) and 22 typically developing children (aged from 8 to 12 years old; 2 female) participated in this study. All of the participants were right-handed, which was confirmed using the Edinburgh Handedness Inventory (Oldfield, 1971) in the TDC cohort and by oral report in the healthy adult cohort. All of the TDC were recruited as controls for ongoing studies of ASD and ADHD. In TDC, the Wechsler Intelligence Scale for Children Third or Fourth Edition (WISC-III/IV) was used to assess intellectual ability. Children with full-scale IQ scores below 80 were excluded from participation (average IQ 114.5 ± 11.6). All of the children in the TDC cohort were free of criteria for psychiatric disorders as tested by the Diagnostic Interview for Children and Adolescents-Fourth Edition (DICA-IV), and none of the children in the TDC cohort were prescribed psychoactive medications. Informed consent was obtained from adult subjects and a parent of each child (who themselves assented to testing), under the approval of the Kennedy Krieger Institute and The Johns Hopkins School of Medicine Institutional Review Boards.

### 2.2. Stimulus delivery

A CM4 four-digit tactile stimulator (Cortical Metrics) was used for stimulation (Holden et al., 2012). All of the stimuli were delivered to the glabrous skin of the left digit 2 (LD2) and digit 3 (LD3) using a cylindrical probe (5 mm in diameter), and all stimuli were in the flutter range (25–50 Hz). Visual feedback, task responses, and data collection was performed using an Acer Onebook Netbook computer, running CM4 software (Holden et al., 2012).

### 2.3. Experimental design

**Fig. 1:**
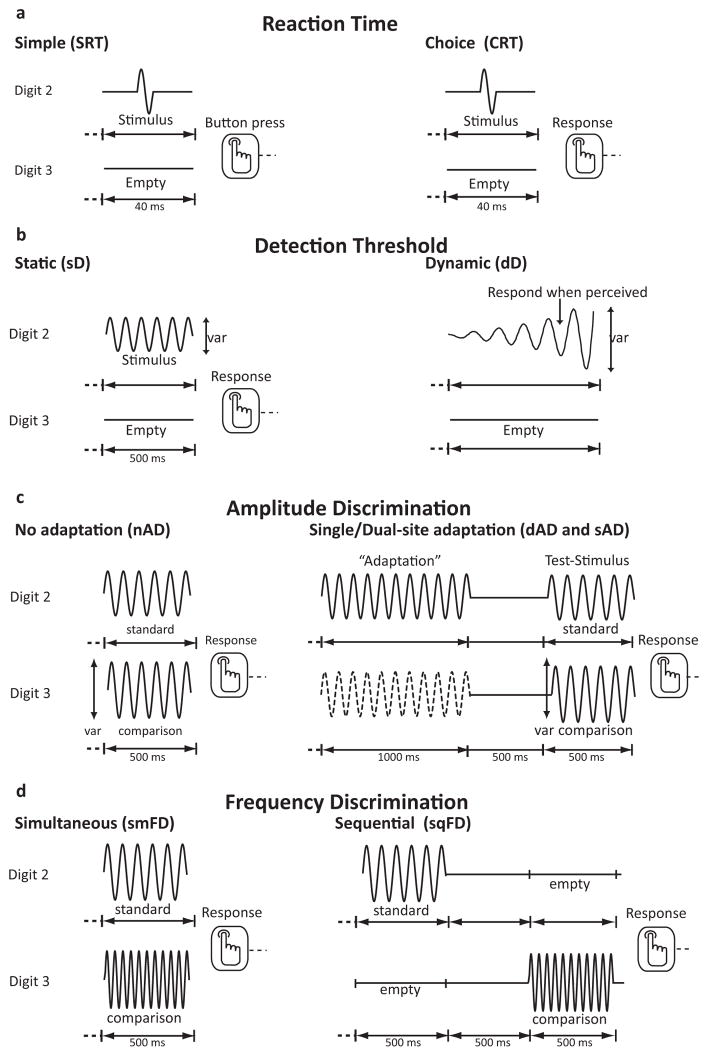
Vibrotactile testing battery, trial examples. (a) Simple (sRT) and choice (cRT) reaction time. (b) Static (sD) and dynamic (dD) detection threshold. (c) Amplitude discrimination without adaptation (nAD), with dual-site adaptation (dAD) and single-site adaptation (sAD). (d) Sequential (sqFD) and simultaneous (smFD) frequency discrimination.

The vibrotactile testing battery consisted of nine tasks, as shown in schematic form in Fig. 1. Prior to each task, the participants had to correctly respond to three consecutive practice trials to proceed, to confirm that the subject understood the instructions. Feedback was given during the practice trials but not during the task trials. In all tasks, stimulus delivery was pseudo-randomized between LD2 and LD3. The response was obtained via a mouse-click using the participant’s right hand. The left mouse button corresponded to LD3 and the right mouse button to LD2. All of the data were visually inspected prior to analysis.

#### 2.3.1. Reaction time: simple (sRT) and choice (cRT) reaction time

A suprathreshold stimulus (frequency 25 Hz, amplitude 300 μm, duration 40 ms) was delivered on LD2 or LD3, and the participants were asked to respond as quickly as possible when they felt the stimulus. In the sRT task, a mouse-click was sufficient, whereas in the cRT task, the participants additionally had to determine on which finger they felt the stimulus (inter-trial interval (ITI) 3 s; 20 trials). For each individual, the reaction times (for correct trials only in the cRT task) were sorted in ascending order, and the mean of the median 6 values was obtained as the mean RT.

#### 2.3.2. Detection threshold: static (sD) and dynamic (dD) detection threshold

In the sD task, a supra-threshold stimulus (frequency 25 Hz, starting amplitude 25 μm, duration 500 ms) was delivered to either digit and the participants were asked to respond on which finger they felt the stimulus. A 1 up/1 down tracking paradigm (stimulus amplitude was decreased for a correct answer and increased for an incorrect answer) was used for the first 10 trials and a 2 up/1 down (two correct answers were necessary for a reduction in test amplitude) was used for the remainder of the task (ITI 5 s; 24 trials). The sD threshold was obtained as the mean amplitude of the final four trials, and the amplitude was determined for the twenty-fifth trial. In the dD task, after a variable delay (0–2500 ms), a 25 Hz stimulus increased from zero amplitude (rate of amplitude increase 2 μm/s). The participants were asked to respond as soon as they felt the stimulus and to indicate the finger on which the stimulus was felt (ITI 10 s; 7 trials). The DD threshold was obtained as the mean stimulus amplitude at the time of pressing the button, across all correct trials.

#### 2.3.3. Amplitude discrimination threshold with no adaptation (nAD), dual-site adaptation (dAD) and single-site adaptation (sAD)

The amplitude discrimination tasks have been previously described (Tannan et al., 2007b; Tommerdahl et al., 2008a). In the nAD task, the participants were asked to choose which of the two simultaneously delivered stimuli had the higher amplitude (25 Hz; 500 ms; Standard stimulus amplitude: 100 μm; initial comparison stimulus amplitude: 200 μm). A 1 up/1 down tracking paradigm (comparison stimulus amplitude was decreased for a correct answer and increased for a wrong answer) was used for the first 10 trials and a 2 up/1 down (two correct answers were necessary for a reduction in comparison stimulus amplitude) was used for the remainder of the task (ITI 5 s; 20 trials). In the dAD condition, each trial was preceded by dual-site-delivered adapting stimuli (25 Hz; duration 1 s, amplitude 100 μm) and in the sAD task, each trial was preceded by a single-site-delivered adapting stimulus (duration 1 s, amplitude 100 μm). Amplitude discrimination thresholds were obtained as the mean amplitude of the final four trials, and the amplitude was determined for the twenty-first trial.

#### 2.3.4. Frequency discrimination threshold: sequential (sqFD) and simultaneous (smFD)

In the sqFD task, stimuli (500 ms; 200 μm) were delivered to LD2 and LD3 sequentially (inter-stimulus interval 500 ms; pseudo-random location). In the smFD task, the two stimuli were delivered simultaneously to both LD2 and LD3 (pseudorandom location). One finger always received the standard stimulus (30 Hz) and the other the comparison stimulus (initial frequency 40 Hz). In both conditions, the participants were asked which finger received the higher frequency stimulus. The 1 up/1 down tracking paradigm (comparison stimulus frequency was decreased for a correct answer and increased for a wrong answer) was used for the first 10 trials and the 2 up/1 down (two correct answers were necessary for a reduction in comparison stimulus frequency) was used for the remainder of the task (ITI 5 s; 20 trials). Frequency discrimination thresholds were obtained as the mean of the frequency of the final four trials, and the frequency was determined for the twenty-first trial. Previous frequency discrimination studies have shown that the perceived intensity varies as a function of frequency as well as intensity (LaMotte and Mountcastle, 1975; Verrillo and Capraro, 1975). However, Harris et al. (2001) previously reported that the “subjects’ accuracy at comparing frequency was not affected by shifts in vibration amplitude that causes the two vibrations to have equivalent intensity (i.e., by increasing the amplitude of a lower frequency)”. In this study, the amplitude was constant for both the standard and comparison stimuli, and the order of higher/lower was randomized across digits.

### 2.4. Analysis

The participants’ data for an individual task were excluded when it was reported – orally by the experimenter- that the participant did not execute the task properly and showed poor behavioral compliance (e.g., pressing buttons as quickly as possible without regard for the stimulus and task), or when inspection of the tracking-profile showed large deviations in stimulus value over the last five trials (greater than four times the starting value, divided by the number of trials). Initial analysis focused on comparisons between related tasks (i.e., paired *t*-test between sRT and cRT; paired *t*-test between sD and dD; paired *t*-test between dAD and sAD; ANOVA of the three AD tasks; paired *t*-test of smFD and sqFD) keeping HA and TDC separate. The correlation matrices were calculated for all nine tasks for the HA and TDC groups independently. A clustering den-drogram was calculated from the normalized correlation matrix to investigate the relationships between the different tasks.

## 3. Results

One participant was fully excluded from the TDC cohort due to poor execution of all tasks, based on an oral report by the experimenter and confirmed visual inspection of data.

### 3.1. Reaction time

**Fig. 2:**
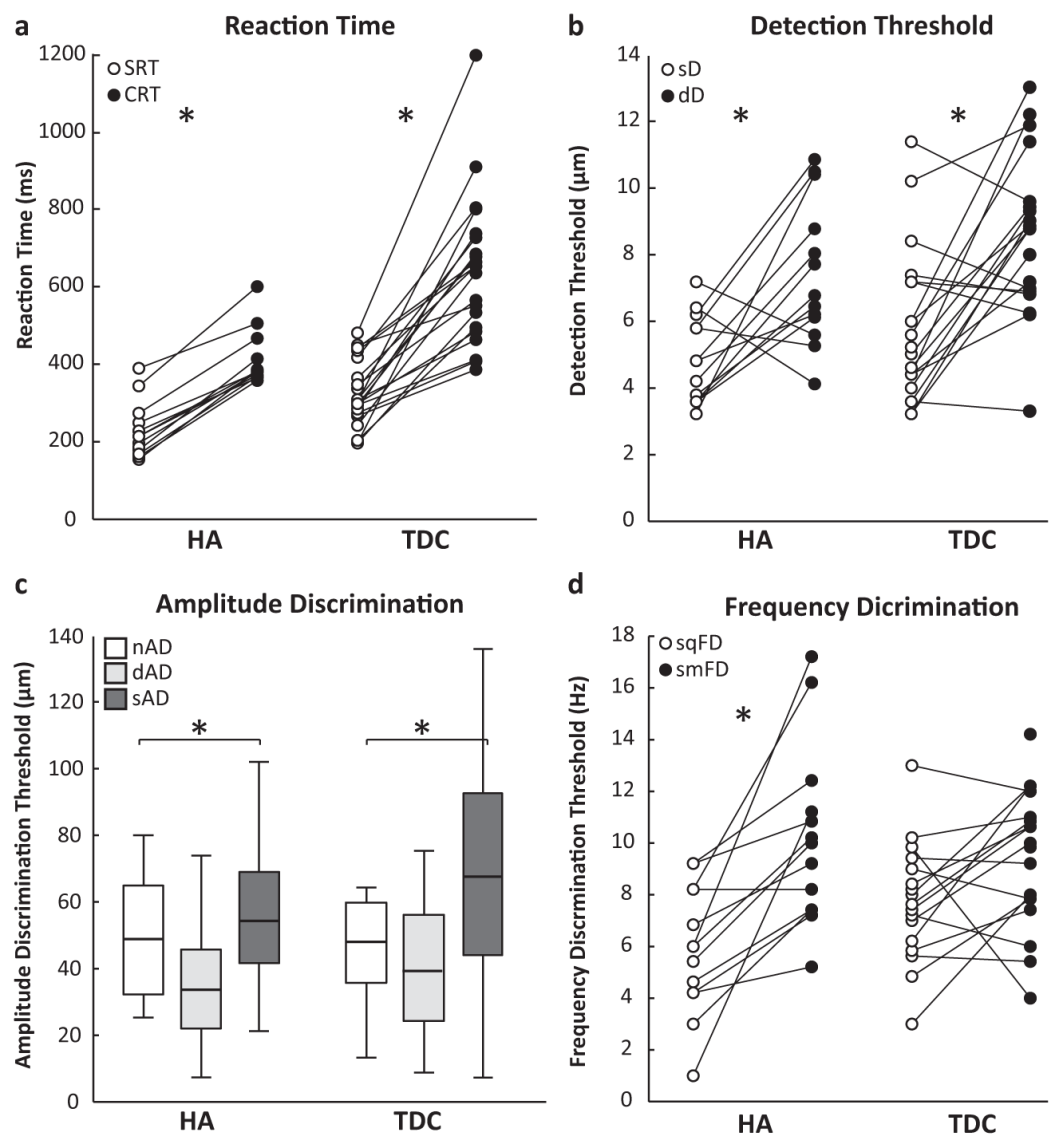
Individual results for all tasks. (a) Reaction time. RT was faster in the sRT task than in the cRT task in both HA and TDC (*p* < 0.001). The TDC were significantly slower (*p* < 0.01) than the HA. (b) Detection threshold. The sD was significantly lower than the dD in both HA and TDC (*p* < 0.001). There was no significant difference in the detection threshold between HA and TDC. (c) Amplitude discrimination. In HA, the sAD threshold was significantly worse than the dAD (*p* < 0.02) and close to significance from nAD (*p* = 0.0494, uncorrected for multiple comparisons). The dAD was close to being significantly different from nAD (*p* = 0.055). In TDC, the sAD was also significantly worse than the dAD and nAD (*p* < 0.02), but the dAD and nAD did not differ significantly. There were no differences between the cohorts. (d) Frequency discrimination. sqFD was significantly better than smFD in HA (*p* < 0.05), but not in TDC. *p < 0.05. Box plot whisker are 5th and 95th percentile, center of the box is the *mean*.

The mean RT for the sRT task and cRT tasks were 227.03 ± 71.61 ms and 411.2 ± 71.07 ms, respectively, for the HA group (an average increase in RT of 90% between the sRT and cRT) and 320.72 ± 84.51 and 640.6803 ± 191.60 ms for the TDC (an average increase in RT of 108% between the sRT and cRT), as shown in Fig. 2a. As expected, the mean RT was slower for the cRT task than for the sRT tasks for both groups (paired *t*-test *p* < 0.0001). The RT was significantly slower in both tasks for TDC compared to HA (*p* < 0.01 for both tasks), although the increase in RT was not.

### 3.2. Detection threshold

For the HA group, the mean sD threshold was 4.84 ± 1.3 μm and the mean dD threshold was 8.4 ± 2.05 μm. For the TDC group, the mean sD threshold was 5.75 ± 2.28 μm and the mean dD threshold was 8.68 ± 2.35 μm. As the stimulus amplitude continued to increase between perception and response, the reaction time component in the dD task increased its threshold values; thus, the dD threshold for each individual was corrected using their mean cRT and the rate of amplitude increase. This resulted in corrected dD thresholds of 7.44 ± 2.16 μm for the HA group and 7.42 ± 2.32 μm for the TDC group. The sD and corrected dD are shown in Fig. 2b. The dD threshold was significantly greater than the sD threshold in both groups (*p* < 0.001). The results from one additional participant in the TDC group were excluded due to poor execution of the task. There were no differences in task performance or difference in sD–dD difference between the two cohorts (*p* > 0.5).

### 3.3. Amplitude discrimination

In the HA group, the mean AD threshold was 46.15 ± 18.5 μm without adaptation, 34.38 ± 18.57 μm with dual-site adaptation (an average increase in performance of 21%) and 58.08 ± 21.84 μm with single-site adaptation (an average decrease in performance of 36%), as shown in Fig. 2c. A one-way ANOVA showed a significant difference between the task performance in the three adaptation conditions, and post hoc paired *t*-tests showed that the dAD and sAD thresholds were significantly different (*p* = 0.013) and that the differences between dAD and nAD and between sAD and nAD were close to threshold (*p* = 0.055, and *p* = 0.0494, respectively).

For the TDC group, the mean AD threshold was 49.21 ± 29.98 μm without adaptation, 39.41 ± 22.20 μm with dual-site adaptation (an average increase in performance of 20%) and 65.81 ± 36.22 μm with single-site adaptation (an average decrease in performance of 34%). A one-way ANOVA showed a significant difference (*p* < 0.05) between the conditions, and post hoc paired *t*-tests showed that both nAD and dAD thresholds were significantly different from the sAD thresholds (*p* = 0.010, and *p* = 0.001, respectively) but that they did not differ from one another (*p* = 0.7). Four TDC participants were excluded from the amplitude discrimination task (one for nAD, three for dAD) due to poor execution of the task and improper tracking. Moreover, there were no significant differences in AD performance between the two cohorts (*p* = 0.08 for nAD, *p* > 0.5 for dAD and sAD).

### 3.4. Frequency discrimination

For the HA group, the mean frequency discrimination threshold was 5.4 ± 2.4 Hz in the sequential condition and 10.2 ± 3.5 Hz in the simultaneous condition (significantly different at *p* < 0.0001), as shown in Fig. 2d. For the TDC group, the mean frequency discrimination threshold was 7.68 ± 2.31 Hz in the sequential condition and 9.31 ± 2.61 Hz in the simultaneous condition (not significantly different at *p* < 0.05). In the TDC group, one participant was excluded for both tasks due to poor execution, three were excluded for the sequential condition due to poor tracking, and one was excluded from the simultaneous task due to poor tracking. The TDC performed significantly worse than the adults in the sequential, but not in the simultaneous FD task (*p* < 0.05, *p* > 0.4, respectively).

### 3.5. Correlation analysis of all tasks

**Fig. 3:**
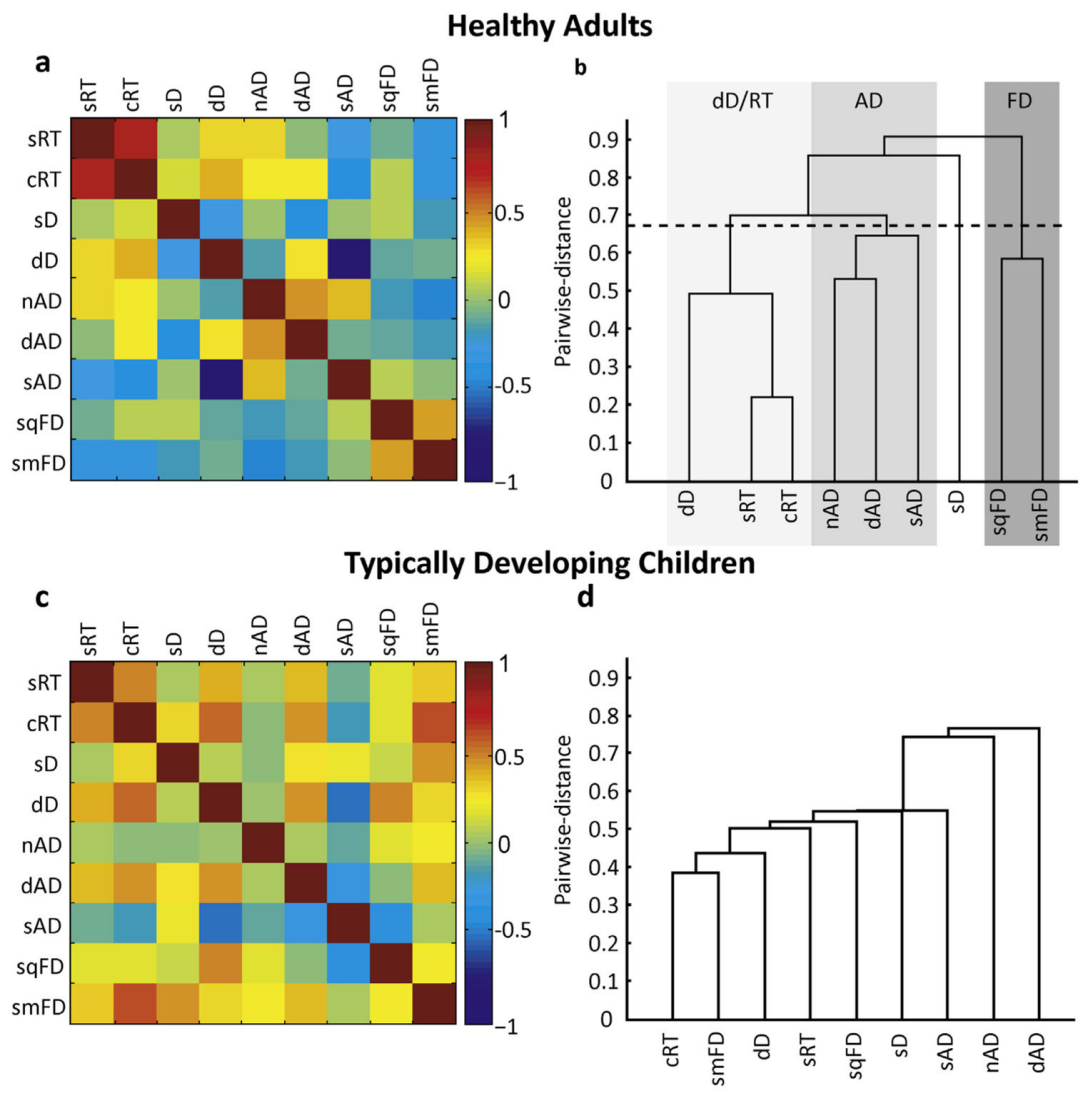
Correlation matrix and cluster analysis. (a) In HA, three different task groupings (RT + DT; AD; FD) correlated with each other. Furthermore, the sAD was negatively correlated with the corrected dD threshold (*R* = −0.83). (b) Cluster analysis clustered different tasks-groups within separate branches, although dD clustered with RT to some extent. (c) In the TDC, the correlation matrix showed more, but weaker correlations. The RT tasks are correlated with each other (*R* = 0.49) and with both detection threshold tasks (*R* = 0.38). Consistent with the HA group, the dD threshold was negatively correlated with the sAD (*R* = −0.51). However, the correlations among the amplitude discrimination tasks and among the frequency discrimination tasks, as shown in the HA group, were absent in the TDC group. (d) No clustering could be observed in the TDC.

The correlation matrix for the HA group, shown in Fig. 3a, demonstrates particularly strong relationships between the two RT tasks, between the FD tasks, and between the three amplitude discrimination tasks. Furthermore, the amplitude discrimination following single-site adaptation was negatively correlated with corrected dynamic detection threshold (*R* = −0.83). There was a correlation between the dD threshold and cRT, which decreased after correcting for reaction time, suggesting an important role of reaction time in the dD threshold (*R* = 0.5 and 0.38, respectively). The clustering dendrogram in the analysis depicted in Fig. 3b showed that the related tasks clustered together (RT, *R* = 0.78; AD, *R* = 0.35–0.46; FD, *R* = 0.42), although the dynamic detection threshold (both corrected and uncorrected dD) clustered to some extent with the RT tasks. The correlation matrix for the TDC group, as shown in Fig. 3a, appeared to show more, but weaker, correlations. Similar to the HA group, the RT tasks were correlated with each other as well (*R* = 0.49) and with both detection threshold tasks (*R* = 0.38). Consistent with the HA group, the dynamic detection threshold was negatively correlated with amplitude discrimination following single-site adaptation (*R* = −0.51). However, the correlations among the amplitude discrimination tasks and among the frequency discrimination tasks as shown in the HA group were absent in the TDC group, and the dendrogram appeared uninformative.

## 4. Discussion

We have presented vibrotactile behavioral data on a battery of tasks that can be collected from cohorts of healthy adults (HA) and typically developing children (TDC) in a total testing time of approximately 30 min. Both HA and TDC of 8–12 years of age were able to perform these tasks. Moreover, we have shown not only that TDC are able to perform these tasks but also that they show the same patterns of performance as healthy adults (the effect of feed-forward inhibition on detection threshold, the effect of adaptation on amplitude discrimination and the effect of synchronous stimulation on frequency discrimination), which suggested that the mechanisms underlying these tasks were similar between healthy adults and healthy children. It should be noted that three adult female participants performed the tasks. It is possible that menstrual cycle has an effect on tactile sensitivity, although the evidence is inconsistent (Bajaj et al., 2001, 2002; Drobek et al., 2002). All of the participants performed the tasks within a single session, and any differences in sensitivity due to hormonal effects were expected to be reflected within the variance of the data.

In both HA and TDC, as expected, the choice reaction times were longer than the simple reaction times, and the two results were highly correlated across individuals within both groups. Children performed significantly more slowly than adults in both reaction time tasks, consistent with previous studies showing a U-shaped relationship between age and reaction time (Williams et al., 2005).

The static detection threshold was slightly higher for TDC, which was consistent with previous findings (Bernstein et al., 1986; Guclu and Oztek, 2007), although it was not significant. There was no significant difference between groups in the dynamic detection threshold and no difference at all after correction for reaction time. It has previously been shown that the dD threshold was greater than sD threshold in HA and that adults become worse at both tasks with age (Zhang et al., 2011). To the best of our knowledge, this task has not previously been examined in children. The difference between the dynamic and static detection thresholds has been suggested to be related to feed-forward GABAergic inhibition. Favorov and Kursun (2011) have suggested that layer IV is highly involved in computations of the feed-forward inhibitory drive, which is affected by prior stimulus information. Blankenburg et al. (2003) showed that a preliminary sub-threshold stimulus 30 ms prior to the detection trial increases the detection thresholds, and the authors proposed that this effect was due to cortical feed-forward inhibitory mechanisms, as inhibitory interneurons have a lower spiking threshold than excitatory neurons and are therefore more strongly activated by sub threshold stimuli (Gil and Amitai, 1996). Blankenburg et al. (2003) discussed the possibility that this feed-forward inhibition might be protective against spurious activity in the cortex by decreasing the net cortical activity. Zhang et al. (2011) suggested that sub-threshold stimulation in the dynamic threshold task drives inhibitory mechanisms, which was supported by the observation that the difference diminishes with age (Zhang et al., 2011) as GABAergic inhibition declines. It also appears that this feed-forward processing was fully developed in the cohort examined (8–12 years old).

In amplitude discrimination, dual-site adaptation tends to improve performance, whereas single-site adaptation worsens it, as expected from previous studies (Tannan et al., 2007a,b; Zhang et al., 2011). Our results were consistent with (although extrapolatory to) previous reports that amplitude discrimination performance and the effect of adaptation did not change with age (Zhang et al., 2011).

In frequency discrimination, both cohorts performed worse in the simultaneous frequency discrimination task. In fact, most subjects had significant difficulty in advancing beyond the practice trials and reported being unable to perform the task (some children were unable to perform either task, supported by an oral report from both children and experimenters, potentially due to frequency as an abstract concept). The mean simultaneous FD threshold was not significantly different from the initial difference of 10 Hz. It appeared that whereas sequentially applied stimuli can be distinguished on the basis of frequency, simultaneously applied stimuli cannot, or at least not better than 10 Hz. It is possible that synchronization between cortical representations of the two stimuli, mediated by lateral inhibitory pathways, reduced the perceptual separation of signals and impaired task performance. The detrimental effect of cortical synchronization between digits on stimulus separation has been previously described (Tommerdahl et al., 2008), with reference to temporal order judgment. In that study, negative effects of synchronization were not observed in participants with autism, and the authors suggested this phenomenon could be due to reduced GABAergic local connectivity.

The percepts of frequency and amplitude are not independent, and higher frequencies tend to be perceived as having higher amplitude (LaMotte and Mountcastle, 1975; Verrillo and Capraro, 1975). Some studies (Goble and Hollins, 1994) have used frequency-amplitude matching to remove amplitude as a potential driver of frequency discrimination performance (also; Goble and Hollins, 1994). The aim of this study was to develop a short battery of tasks. Thus, we used a frequency discrimination task that involved physically equal rather than perceptually matched stimulus amplitudes, as has been done by Harris and colleagues (e.g., (Harris et al., 2001, 2006)). Harris et al. (2001) found that amplitude matching did not reduce frequency discrimination performance, and interestingly, our current results showed no significant correlation between amplitude and frequency discrimination performance. One potential improvement on physically matched amplitudes would be to introduce an amplitude jitter.

Interestingly, the correlational and clustering analysis in the HA group tended to sort tasks by property. Thus, the subjects who performed well at the simple reaction time task tended to perform well at the choice reaction time; subjects who performed well at the static detection threshold task also tended to perform well at the dynamic task; etc. This outcome was perhaps not surprising; however, the fact that the two frequency discrimination tasks clustered suggested that it was not true to simply imply that the subjects ‘cannot perform the task.’ The modulus mean between-task correlation coefficient was 0.23 for HA and 0.26 for children (compared to the maximum correlation value of 0.78 in HA), suggesting that it was not true that these tasks were equivalent and that the subjects did not perform ‘well’ or ‘badly’ in equal measure across tasks. This finding was the main value of performing a battery of tasks such as the one presented. In general, the clustering of similar tasks suggested that measuring performance with respect to different properties of vibrotactile stimuli targeted different aspects of cortical processing.

The correlational analysis did reveal some links between task groupings. The relationship between detection threshold and reaction time was not expected, although both tasks were related to white matter integrity (Tamnes et al., 2012; Kerchner et al., 2012). The negative correlation between detection threshold and amplitude discrimination (non-adapted and with single-site adaptation) makes sense in terms of cortical inhibition; subjects with greater levels of inhibition will have higher detection thresholds but would be expected to show better discrimination. Interestingly, this relationship was not maintained for dual-site adaptation, although this result could be due to a bottom effect and there might not be room for improvement in the dual-site adaptation condition. In addition, in neither HA nor TDC were frequency and amplitude discrimination correlated, which may provide additional evidence that amplitude information is not used in the frequency discrimination task.

Correlation and dendrogram analyses revealed differences in the way the domains are related between HA and TDC. However, neither the frequency tasks nor the amplitude discrimination tasks appeared to be correlated in the TDC, while they were in HA. Differences in task relationships between HA and TDC may be indicative of development (neither cutaneous nerves nor spinal cord mature fully until after puberty (e.g., (Allison et al., 1984; Sato et al., 1977)), sensorimotor development, or the development of attentional control. It is possible that some children have more difficulty with tasks than others, thereby increasing variability and masking correlational relationships.

### 4.1. Limitations

While it is beneficial to present a battery of tasks to investigate a number of different processes, these methods do have some limitations. The aim of this battery was that it could be performed within 30 min, which makes it suitable for a naïve cohort as well as for pediatric populations. However, most behavioral tasks described in the psychophysics literature are lengthy (in terms of trial numbers and task repeats), and it remains unclear what effect our shorter protocols have on the accuracy of the measurements. However, the brevity of testing does allow for a much greater number of participants to be tested at the same time, partially offsetting any loss of statistical power.

The task duration was reduced by starting the tasks with relatively difficult initial settings, simultaneous presentation of stimuli to two digits (i.e., smFD is shorter than sqFD), and the randomization of the order of stimuli and parameters not being tested (e.g., amplitude was pseudo-random in the frequency discrimination tasks to reduce discrimination on the basis of amplitude, without having to perform frequency–amplitude correction). Visual inspection of the tracking curves showed that the majority of the participants reached a plateau. The threshold was obtained as the mean of the final five trials, and the average coefficient of variation in adults for all tasks was less than 10% of the average threshold value, indicating a small variability of the last five trials. In addition, the results shown by our naïve cohort compared well with previous findings on these tasks. Testing a pediatric cohort can be challenging, and increasing the number of trials, while potentially increasing the SNR of the measurement, would be expected to adversely affect compliance and threshold measurements. Testing the reproducibility of these measurements within naïve cohorts is problematic because a number of different studies have shown effects of perceptual learning in these tasks. However, the strong within-task-group correlations observed are at least circumstantial evidence for good within-task reproducibility.

In conclusion, we have presented a 30 min tactile behavioral battery that probes a number of different cortical mechanisms and that is easily applied to adults and children as young as 8 years old.

## Abbreviations

HA: healthy adults
TDC: typically developing children
LD2/LD3: left digit 2 and left digit 3
sRT: simple reaction time task
cRT: choice reaction time task
sD: static detection threshold task
dD: dynamic detection threshold task
nAD: amplitude discrimination – no adaptation
dAD: amplitude discrimination – dual-site adaptation
sAD: amplitude discrimination – single-site adaptation
sqFD: sequential frequency discrimination
smFD: simultaneous frequency discrimination
ISI: interstimulus interval
ITI: intertrial interval
